# Temperature dependent in vitro binding and release of target DNA by Cas9 enzyme

**DOI:** 10.1038/s41598-022-19485-x

**Published:** 2022-09-09

**Authors:** Serene Rose David, Sumanth Kumar Maheshwaram, Divya Shet, Mahesh B. Lakshminarayana, Gautam V. Soni

**Affiliations:** Raman Research Institute, Bangalore, Karnataka 560080 India

**Keywords:** Biochemistry, Biological techniques, Biophysics

## Abstract

The CRISPR-associated protein 9 (Cas9) system has proven to be a powerful technology for genome editing in a wide variety of in vivo and in vitro applications. CRISPR–Cas9, when loaded with the guide RNA, cleaves the DNA at the target position as recognized by the guide RNA sequence. For successful application of this technology, it is important to study the biophysical parameters affecting its function. Temperature dependence of the Cas9 binding as well as energetics of product release after cleavage has not been well reported in the literature. In this work, we study the binding properties of Cas9 enzyme to the sequence specific target DNA at a range of temperatures and, surprisingly, find that the Cas9 enzyme, in our study, can find and bind its target DNA with 90 ± 20% efficiency at temperatures as low as 4 °C. Further, we show that the cleaved DNA products remain bound to the Cas9 enzyme strongly and is released from the enzyme only at higher temperatures. Using the gel shift assays, we quantify the rate of Cas9 binding to target DNA to be 0.8 ± 0.2 min^−1^ at 37 °C. We also tested denaturant (SDS) dependent release of cleaved product which showed a similar release pattern with a dissociation constant of 0.23 ± 0.04 mM. Our results of heat and denaturant dependence on Cas9–DNA binding and release mechanics will provide valuable insights for developing temperature dependent applications of the CRISPR–Cas9 technology.

## Introduction

The CRISPR (Clustered Regularly Interspaced Short Palindromic Repeats)–Cas9 nuclease system is a powerful tool for genome editing due to its efficient targeting of specific sequences in the genome^1^. Cas9 is a relatively large protein (~ 158 kDa) with a bi-lobed architecture consisting of the REC and NUC lobes^2,3^. The NUC lobe contains the HNH and RuvC domains as well as a C-terminal domain (CTD) which interacts with the protospacer adjacent motif (PAM) site^2,4^ on the target DNA. In bacteria, transcription of the CRISPR array followed by enzymatic processing yields short CRISPR RNAs (crRNAs) that directs Cas9 enzyme to target certain sections of the viral genome that prevents the virus from carrying out its normal function^5–7^. This CRISPR–Cas9 technology of DNA targeting and editing is being explored for a wide range of applications from agriculture for designing better quality grains and fruits to human health by developing new treatment techniques and gene therapy^8,9^. Typically, Cas9 enzyme binds to a guide RNA (a duplex of crRNA and *trans*-activating crRNA (tracrRNA), referred to as RNA in the rest of this text). This RNA loaded Cas9 enzyme then finds the target sequence on the template DNA and cuts the double stranded DNA. The binding specificity of Cas9-RNA complex to DNA is important for genome engineering applications. Previously, the effects on the binding properties of Cas9 to the targets when mismatches are introduced in its proximal and distal ends of the PAM sites have been reported^10,11^. The role of PAM sequence in the target recognition and cleavage had also been studied by generating mutations in the PAM site on the complementary or non-complementary strands, or both^12^. Although the specificity of Cas9 to recognise and cleave the target largely depends on the availability of PAM site and its upstream sequences, the binding and cleavage efficiency of Cas9 largely depends on the spacer length and complementarity^13^. The kinetic characterization of enzyme has been performed using stopped-flow and quench-flow techniques and it revealed that DNA cleavage is a two-step process^14^. Cas9 conformational dynamics has been studied by MD simulations and recently some experimental observations were made from NMR experiments^15,16^. Various studies have been reported on the thermostable Cas9 (GeoCas9) isolated from thermophilic bacterium which can survive up to 70 °C^17^ and it was found to have an alternative mechanism for DNA cleavage activity^18^. The temperature dependence of CRISPR–Cas9 mediated genome editing and the effect of different incubation temperatures on cell lines was demonstrated using different genetic tools like ZFN, TALEN, CRISPR and their efficiencies were compared^19^. It has been reported that the Cas9 can remain attached to the cleaved DNA ends in vitro from fluorescence^20,21^ and AFM^22^ studies. There are reports on temperature dependent inhibition in vitro by anti-CRISPR proteins that prevent the binding of Cas9 to its target as well as temperature and light switchable Cas9 variants that can regulate the transcription in a temperature dependent manner, but as per our knowledge, direct measurement of temperature dependence of Cas9 binding and the post-cleavage release of DNA in an in vitro system, is not extensively reported^23,24^. In this work, we present a detailed in vitro study of the temperature dependence of Cas9 binding to its target DNA as well as temperature and denaturant dependent release of the Cas9 cleaved DNA products. From the rate constants measured in this study, we present our understanding of the binding and release model for the Cas9 enzyme.

## Materials and methods

The Cas9 Nuclease, *S. pyogenes* (MO386S) for the experiments was purchased from New England Biolabs (NEB) and the target DNA sequence used in this study was chosen on the supercoiled plasmid pGEM-3z/601 (3025 bp) (gift from Cees Dekker lab, TU Delft). This plasmid was transformed into, amplified and purified from *E. coli DH5α* bacterial strain grown at 37 °C overnight at 180 rpm under ampicillin antibiotic pressure. Nicked circular and linear forms of this plasmid were generated by nicking the plasmid with Nt.BspQ1 (NEB, R0644S) and digesting with ScaI (NEB, R3122S) or NotI (NEB R0189S) enzymes, respectively. The target sequence on the plasmid used in this study is: 5′-GGCACCGGGATTCTCCAGGG-3′. The corresponding CRISPR RNA (crRNA) and *trans*-activating crRNA (tracrRNA) were purchased from Integrated DNA Technology (IDT, USA) and were resuspended in nuclease free buffer (30 mM HEPES, 100 mM potassium acetate, pH 7.5, provided by manufacturer) to make 100 µM stock of each RNA. The 1 kb DNA ladder (N3232S) purchased from NEB was used as size standard for all the gel electrophoresis experiments. Note that the Cas9 and the duplex guide RNA are labelled in short forms as C9 and R in all the gel images.

### Duplex RNA formation

The crRNA and tracrRNA were mixed in nuclease free buffer at an equimolar concentration to prepare a final duplex guide RNA concentration of 1 µM. The mixture was heated to 90 °C for 30 s followed by slow cooling to room temperature (1 h) to make the crRNA-tracrRNA duplex (RNA).

### Cas9-RNA and DNA binding reaction

The RNA was loaded on to the Cas9 enzyme by mixing them in 1:1 molar ratio at 50 nM concentration and incubating for 15 min at 37 °C in cleavage buffer (20 mM Tris–HCl, 100 mM KCl, 5 mM MgCl_2_, 5% glycerol, 1 mM DTT, pH 7.5). Binding to target was achieved by adding the target DNA to the loaded Cas9 in the molar ratio of 1:50:50 (DNA:Cas9:RNA at 1 nM:50 nM:50 nM) and incubating for 1 h at 37 °C. We use the reaction of *apo*-Cas9 (Cas9 without RNA) with DNA as a control.

### Time-dependent binding kinetics

After the binding reaction, samples were removed at respective time points t = 0 s, 4 s,10 s, 30 s, 40 s, 50 s, 1 min, 2 min, 3 min, 10 min, 30 min and 60 min into separate tubes containing 25 mM EDTA and incubated on ice for 30 min to stop the reaction.

### Temperature /SDS-dependent product release

Here, after the binding reaction sample was split into two halves. One half was used as binding control at 37 °C and the other half was used for checking release of the cleaved product using heat or denaturants (see text in “Results” section).

### Gel analysis

The reaction results were visualized on 1.5% agarose gel (ran at 90 V for 90 min in 1 × TAE buffer), stained in SyBr Gold and imaged using a UV Transilluminator. The quantification of band intensities was done in ImageJ according to the procedure given in “Methods” section of supplementary file. Full-length raw images of all the gels quantified in this study are included in the supplementary information file. The fitting of the data was performed using OriginLab software.

### AFM imaging

For AFM imaging, mica sheets (71853-15, Muscovite Mica V4 grade, Electron Microscopy Sciences) were first cleaned using methanol and dried under nitrogen. Freshly cleaved mica substrate was incubated for 2 min at room temperature with a 25 µl drop of 0.0005% poly-l-lysine (PLL, P4832, Sigma) solution in MilliQ water. Washed and dried PLL coated mica (PLL-mica) was then used for sample deposition. The samples (DNA or Cas9–DNA complexes) were diluted in 1 × TE buffer (10 mM Tris–HCl, 1 mM EDTA, pH 8) to a final concentration of 1 ng/µl and mixed well. 25 µl of the prepared sample was dropped on PLL-mica and incubated for 10 min at room temperature. The substrate was then washed with MilliQ water and desiccated for 10 min to dry and then imaged. Molecular Imaging AFM (Pico Plus—Pico Scan 3000) was operated in tapping mode in air using TAP190-G probes (190 kHz resonant frequency, 48 N/m spring constant, Budget Sensors) for all images. The system was controlled using PicoView 1.14 software. The imaging was performed over a region of 2 × 2 µm^2^ with a scan speed of 4 µm/s (line scanning rate of 1 Hz) and a resolution of 512 × 512 px^2^ using the high-resolution scanner of 10 µm range.

## Results

### Cas9 binding to target DNA: role of DNA conformation

**Figure 1:**
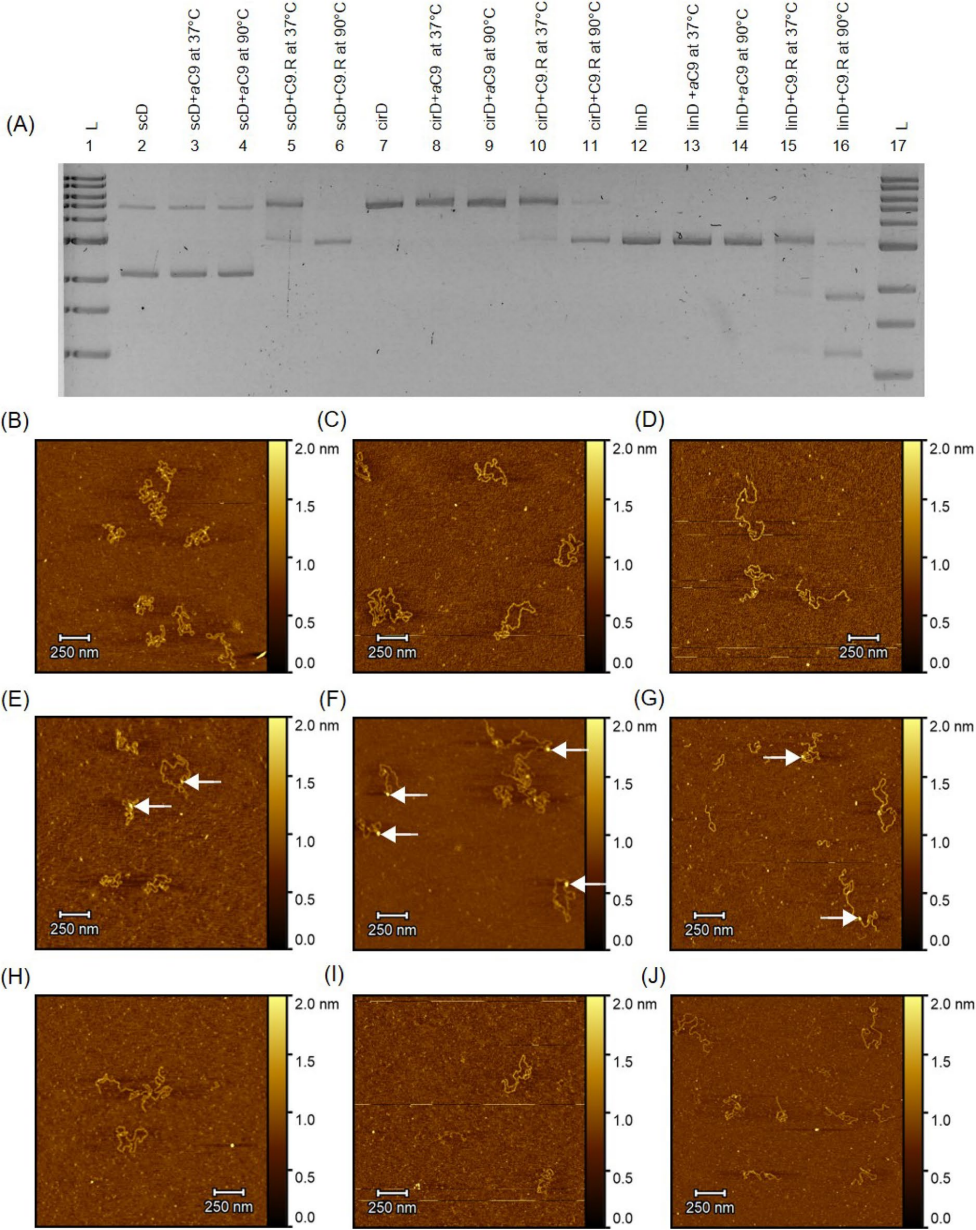
Cas9 binding to different conformations of DNA. (**A**) Gel image showing the Cas9 bound to supercoiled, nicked circular and linear DNA. Lane 1 and 17 show 1 kb ladder. Lanes 2, 7 and 12 show control DNA. Lanes 5, 10 and 15 show the gel shift due to Cas9 bound DNA. Lanes 6, 11 and 16 show the release of cleaved products upon heating. (**B**–**J**) Shows the AFM images of samples scanned using tapping mode in air. The three columns show supercoiled, circular and linearized plasmids respectively. (**B**–**D**) Shows AFM images of bare supercoiled, nicked, and, linearized form of the plasmid, respectively. (**E**–**G**) Shows Cas9-bound (white arrows) states of the three plasmid conformations. (**H**–**J**) Show heat-released products of Cas9 cleavage of the three plasmid conformations.

We first test the changes in electrophoretic mobility upon Cas9 binding to different DNA conformations. In Fig. 1A, we show binding of RNA loaded Cas9 enzyme to target DNA plasmid in three different DNA conformations: supercoiled (scD) (lanes 2–6), nicked circular (cirD) (lanes 7–11) and linear (linD) DNA (lanes 12–16). Note that the Cas9 enzyme without the targeting guide RNA (*apo*-Cas9) does not bind to the target DNA (lanes 3, 8 and 13). For all three DNA conformations, the Cas9 binds to the DNA and shifts the DNA band only in the presence of the sequence specific targeting guide RNA (lanes 5, 10 and 15). Interestingly, we see the maximum gel shift when the Cas9 binds to the supercoiled (scD) conformation of the DNA (lane 5) when compared to the circular and linear conformations of the plasmid (lanes 10 and 15), where the enzyme bound conformation resulted in only a marginal shift in band position. This would indicate that for the more open DNA targets (linear and circular), Cas9 binding only locally affects the DNA conformation without structurally affecting the rest of non-target DNA.

For the much compact conformation of supercoiled DNA, on the other hand, Cas9 binding dramatically affects the electrophoretic mobility. Importantly, we find that the Cas9 enzyme stays bound to the DNA target after cleavage. This Cas9–DNA complex is stable (for all three conformations) and the cleaved products were released only upon disrupting the complex by heating the reaction mixture to 90 °C. This stable complex suggests that, post cleavage, the Cas9 enzyme keeps the cleaved ends of the DNA in a strongly bound complex. As expected, products of Cas9 cleavage of both scD and cirD resulted in the linear conformation (lane 6 and lane 11) and the linear DNA target was cleaved and the products were released as two shorter linear DNA fragments of lengths 1.2 kb and 1.8 kb respectively (lane 16). Note the faint band corresponding to a small amount of circular DNA in supercoiled DNA sample (see lanes 2–4). For clarity, we re-confirmed our results with gel purified sample of the three conformations and the results are shown in Fig. S1. Figure 1B–D shows the AFM images of all the three conformations: supercoiled (scD), nicked circular (cirD) and linear (linD). Figure 1E–G shows the Cas9 bound state of the three conformations before the heat release. We see here the Cas9 protein (white arrows) bound to the DNA molecules post the 1-h incubation. We note that these molecules were not cleaved. Figure 1H–J shows AFM images of the heat released products of Cas9 cleavage for the three DNA conformations. In Fig. 1H,I, we see linearized products of a single Cas9 cleavage of the supercoiled and circular plasmids, respectively. Figure 1J shows the smaller length DNA products from Cas9 cleavage of linear DNA.

### Cas9 binding to target DNA: time dependence

**Figure 2:**
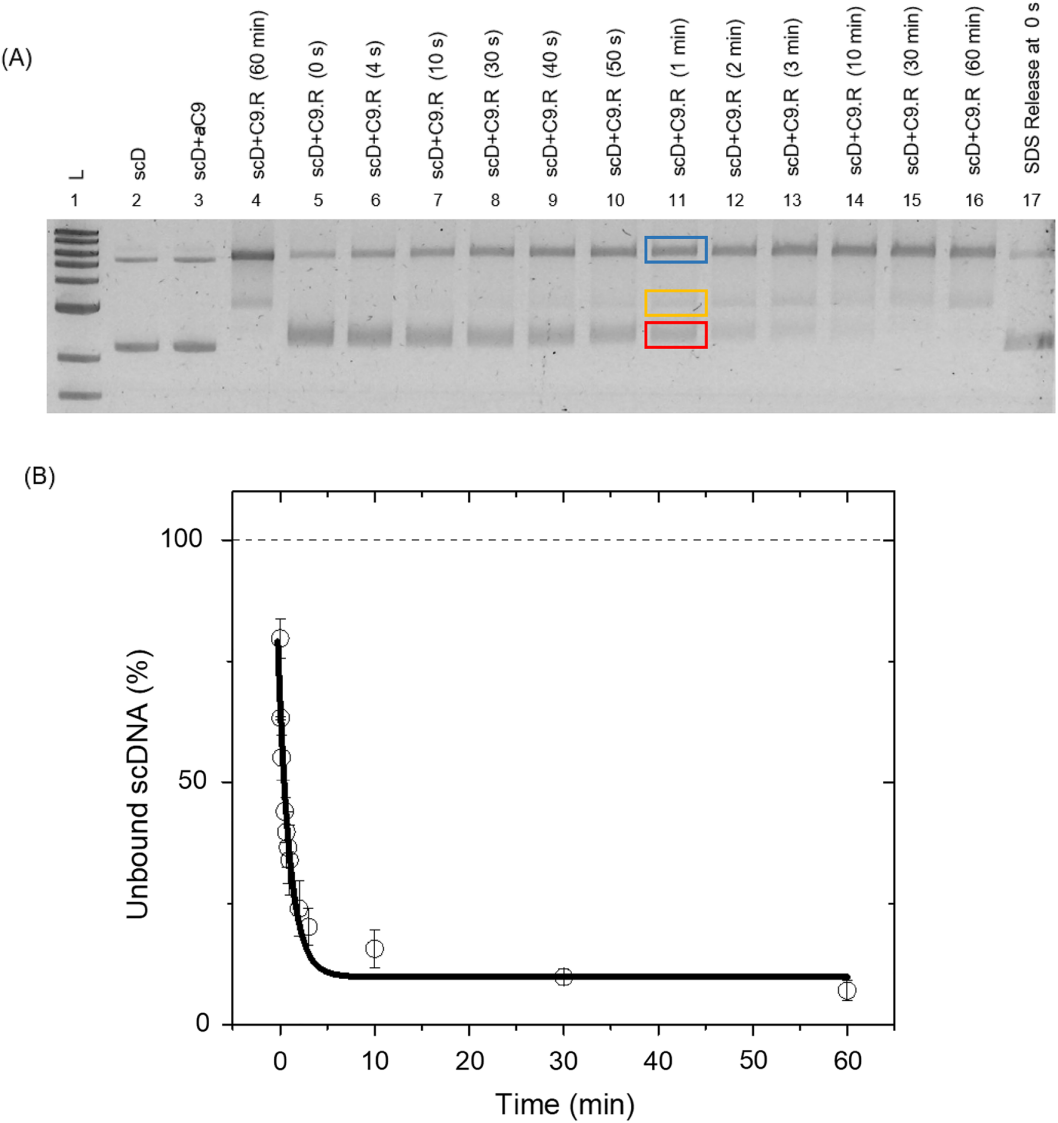
Time dependence of Cas9 binding to target DNA. (**A**) The gel shows the binding of Cas9 to the target DNA as a function of time and the subsequent arrest of the bound product with EDTA. Lane1: 1 kb ladder, lane2: control DNA, lane3: DNA + *apo*-Cas9 at 37 °C, lane4: Cas9.RNA bound to the target DNA at 37 °C for 60 min which is the binding control. Lanes 5–16 show the binding of Cas9.RNA to the target DNA at 0 s, 4 s, 10 s, 30 s, 40 s, 50 s, 1 min, 2 min, 3 min, 10 min, 30 min, 60 min respectively. Lane 17 shows the SDS release of target DNA at 0 s to confirm no linear DNA is released. The blue, yellow and red boxes in lane 11 represent the typical positions for bound, released and the unbound gel bands used for DNA quantification. (**B**) Shows quantification of the Cas9 binding rate constant from the gel band intensities of the unbound supercoiled DNA in lanes 5–16 respectively. (k_bind_ = 0.8 ± 0.2 min^−1^) Solid line is fit to Eq. (1). The mean and error bars are from N = 3 independent experiments (the repeated experiments are shown in Fig. S2).

To measure the binding/release kinetics, supercoiled form of target DNA was used for all further experiments. Target DNA was added to the reaction-mixture with RNA loaded Cas9 and incubated at 37 °C. In order to find the fraction of unbound DNA, 12.5 μl of the sample was collected from the reaction mix at different time points t = 0 s, 4 s,10 s, 30 s, 40 s, 50 s, 1 min, 2 min, 3 min, 10 min, 30 min and 60 min into tubes containing 25 mM EDTA and incubated on ice for 30 min to stop the reaction. One half of the t = 0 s time point sample was used for testing the release of the cleaved product by denaturant (adding 0.29 M SDS for 10 min at 37 °C). The other half was used as control to test for binding at 37 °C for simultaneous visualization on the gel. As seen from Lane 17 in Fig. 2A, absence of any linear DNA confirms that the reaction makes no progress after addition of EDTA to the mixture. Time dependent binding of Cas9 enzyme to target DNA (scD) is shown in lanes 5–16 for the mentioned incubation times respectively.

We found that upon Cas9 binding, the amount of unbound scDNA decreased from 80 ± 4% at 0 s to 7 ± 2% within 60 min (Fig. 2B). We quantify the binding timescales from the gel band intensities in Fig. 2B and calculate the rate constant of the reaction (k_bind_) by fitting data to the following rate equation:

1 $${\text{y}} ({\text{t}} ) = {\text{y}}_{0} {\text{e}}^{{ - {\text{k}}_{{{\text{bind}}}} {\text{t}}}} + {\text{c}}$$

Here, y(t) is the percentage of unbound scDNA as a function of reaction time (t), y_0_ is the initial percentage of unbound scDNA and c is the offset in percentage values. From the fit to Eq. (1), we obtain the rate constant of binding to be k_bind_ = 0.8 ± 0.2 min^−1^.

### Cas9–DNA binding and release of cleaved products: temperature dependence

**Figure 3:**
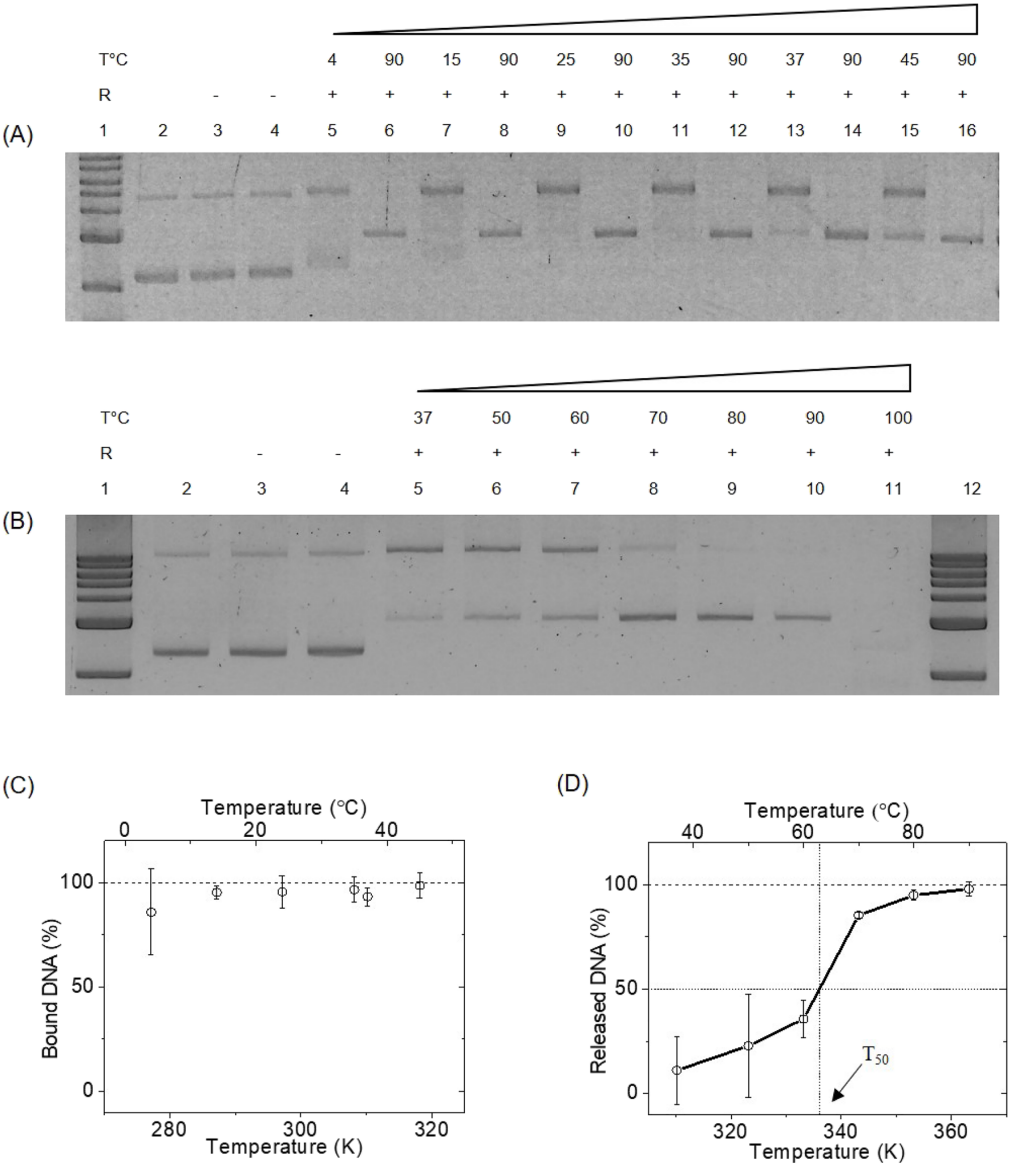
Temperature dependence of Cas9 binding and release of target DNA*.* (**A**) Binding of loaded Cas9 to the target DNA at different temperatures. Lane 1: 1 kb ladder, lane 2: control DNA, lane 3: DNA + *apo-*Cas9 at 37 °C, lane 4: DNA + *apo-*Cas9 at 90 °C, lanes 5, 7, 9, 11, 13 and 15 show the binding of Cas9.RNA to target DNA incubated for 1 h at various temperatures. The lanes 6, 8, 10, 12, 14 and 16 show the release of cleaved products after heating at 90 °C for 10 min. (**B**) Temperature dependent release of cleaved products. Lanes 1 and 12: 1 kb ladder, lanes 2 to 4 is same as that mentioned in (**A**). The lanes 5–10 show the release of cleaved products at temperatures from 37 to 90 °C. (**C**) The percentage of Cas9 bound DNA is estimated from gel band intensities of (**A**) and are plotted for different temperatures. The mean and error bars are from N = 2 independent experiments (see also Fig. S3A). (**D**) The percentage of target DNA released by Cas9 were quantified from gel band intensities and are plotted for different temperatures. The mean and error bars are from N = 3 independent experiments (see also Fig. S3B).

To check the temperature dependence of binding efficiency, we loaded Cas9 with RNA at 37 °C for 15 min and then checked its binding to target DNA at different temperatures. The Cas9 enzyme was incubated with target DNA for 1 h at the following temperatures: 4 °C, 15 °C, 25 °C, 35 °C, 37 °C and 45 °C. After the incubation time, each reaction mix was split into two halves. One half was heated to 90 °C for 10 min to release cleaved products (Fig. 3A: lanes 6, 8, 10, 12, 14 and 16) and the other half was run on gel as binding control (Fig. 3A: lanes 5, 7, 9, 11, 13 and 15). In Fig. 3A, we show binding of Cas9 enzyme to scD at various temperatures. To our surprise, we observe that Cas9 can find and bind to its target DNA with 90 ± 20% (see Fig. 3C) efficiency even at temperatures as low as 4 °C. The enzyme maintained its specific single site cleavage activity towards DNA as evidenced by the correct length of released DNA products in the control lanes.

In our tested range of temperatures, the efficiency of target binding steadily increased at higher binding temperatures and reached saturation with 95 ± 3% at 15 °C as quantified in Fig. 3C.

Next, to investigate the temperature dependent release of the Cas9 cleaved products, we quantified the temperature dependent cleavage assay. In this assay, the RNA loaded Cas9 and target plasmid DNA was mixed and the reaction mixture was incubated for 1 h at 37 °C for the enzyme to bind to the target DNA. This resulted in the formation of Cas9–DNA complex. To release the cleaved products, the reaction mix was then divided into 6 equal aliquots and incubated for 10 min at different temperatures ranging from 50 to 100 °C. Figure 3B shows this temperature dependent release of the cleaved DNA products from Cas9 at temperatures 37 °C, 50 °C, 60 °C, 70 °C, 80 °C, 90 °C and 100 °C in lanes 5–11 respectively. We note a steady decrease in the intensity of the Cas9 bound DNA band and a corresponding increase in intensity of the released products band with increasing temperature. Finally, at 100 °C, we see no sample in the lane, possibly due to sample degradation at high temperatures^25^, as shown in Fig. S4. Almost complete release of cleaved products is attained only at temperatures as high as 80 °C where the enzyme-bound DNA band completely disappears. This suggests a strong binding between the enzyme and its cleaved DNA ends where the product release happens only when the energy supplied from heating exceeds the binding energy or the enzyme denatures. This temperature dependent release dynamics is quantified from the gel band intensities of Fig. 3B and is plotted against temperature in Fig. 3D. From the release dynamics, we find that 50% of the cleaved DNA is released from Cas9 complex at a temperature, T_50_, of 63 °C (336 K) by simple linear interpolation between the data points at 60 °C and 70 °C. This shows that the RNA loaded Cas9 binds to the target specifically, holds the cleaved DNA product tightly until it is forced to release them on increasing the temperature.

### SDS dependent release of cleaved products

**Figure 4:**
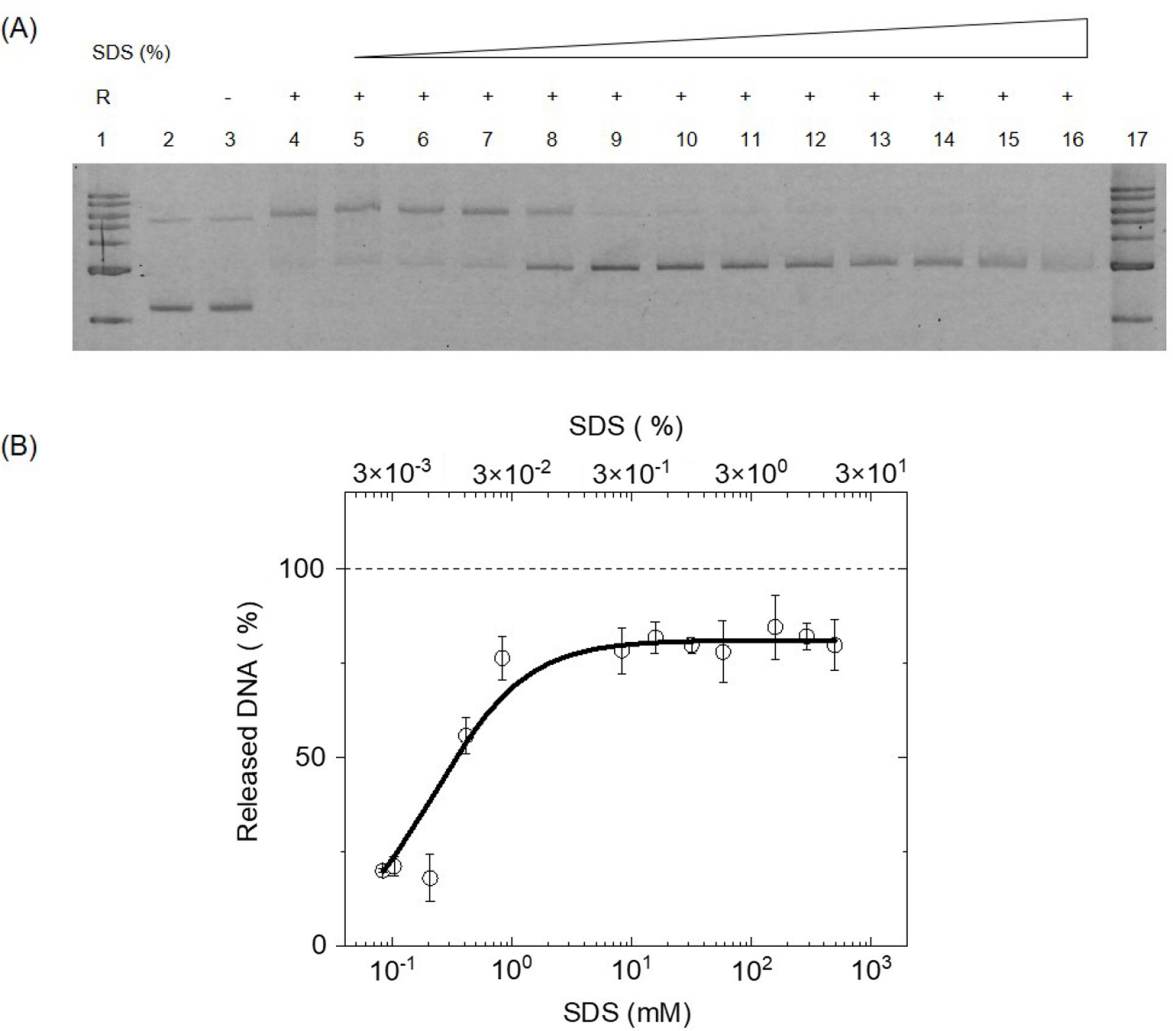
Quantification of SDS dependent release of Cas9 cleaved products. (**A**) Gel image showing the DNA release as a function of SDS %. Lane 1 and 17: 1 kb ladder, lane 2: control DNA, lane 3: DNA + *apo-*Cas9 at 37 °C, lane 4: DNA + Cas9.RNA at 37 °C, lanes 5–16 show the release of cleaved products at final concentrations of SDS—0.083 mM, 0.10 mM 0.21 mM, 0.41 mM, 0.83 mM, 8.3 mM, 16 mM, 32 mM, 58 mM, 0.16 M, 0.29 M and 0.50 M. respectively. (**B**) Plot showing % DNA released as a function of SDS concentration. The mean and error bars are from N = 3 independent experiments (see also Fig. S5). Solid line is fit to Eq. (2).

Finally, we tested systematic release of the cleaved products using a denaturant instead of temperature. With the binding reaction done exactly same as for the temperature case, the reaction mix was then divided into 12 equal parts. Denaturant (SDS) was added to the reaction mixture at a final SDS concentration of: 0.083 mM, 0.10 mM, 0.21 mM, 0.41 mM, 0.83 mM, 8.3 mM, 16 mM, 32 mM, 58 mM, 0.16 M, 0.29 M and 0.50 M. The denaturant releases the cleaved products bound to the Cas9 enzyme in a concentration dependent manner as shown in Fig. 4A.

We can see the increase in the released cleaved DNA products with increase in the SDS concentration and it is quantified in Fig. 4B. Assuming that the denaturant in the reaction mixture directly affects the Cas9–DNA binding, we write the chemical reaction as follows:

$${\text{Cas}}9.{\text{DNA}} + {\text{SDS }} \rightleftharpoons {\text{Cas}}9.{\text{SDS}} + {\text{DNA}}$$

The obtained data can then be modelled with Hill equation shown below:

2 $${\text{y}}( {\text{x}}) = \frac{{{\text{y}}_{{{\text{max}}}} {\text{x}}^{{\text{n}}} }}{{{\text{k}}^{{\text{n}}} + {\text{x}}^{{\text{n}}} }}$$

Here, x is the concentration of SDS (mM) used in the reaction, y(x) is the percentage of bound DNA released as a function of SDS concentration, y_max_ is the saturation percentage of bound DNA released, n is the Hill coefficient, k is the dissociation constant of the reaction or the concentration of SDS at which 50% of the bound DNA is released. From the fit in Fig. 4B, we obtained, y_max_ = 81 ± 2%, k = 0.23 ± 0.04 mM or (6 ± 1) × 10^–3^ (w/v %) and n = 1.1 ± 0.2. The dissociation constant for SDS-induced release of DNA from Cas9 is 0.23 ± 0.04 mM and this is close to the concentration of SDS required to denature the proteins^26^. The requirement of complete denaturation of Cas9 for releasing the DNA also confirms their strong binding to each other.

## Discussion and conclusion

In this work, we present an in vitro study of time dependent binding as well as temperature and denaturant dependent release of Cas9 cleaved DNA products. We first showed time dependent binding of Cas9 to the target DNA at 37 °C and performed the experiment over a range of incubation times from 0 to 60 min. We then quantified the binding timescale from the gel intensities of the bound target obtained immediately after incubation at 37 °C and found that the amount of unbound scDNA decreased from 80 ± 4% to 7 ± 2% within 60 min. The quantification of the unbound scDNA yielded a binding rate constant of 0.8 ± 0.2 min^−1^. The time dependent binding studies showed that the Cas9 binds to the target at a very fast rate and cleaves the target. Moreover, we could understand that SDS has an instantaneous effect on Cas9–DNA binding and can be suggested to stop any Cas9 based reaction in the case of time dependent studies.

**Figure 5:**
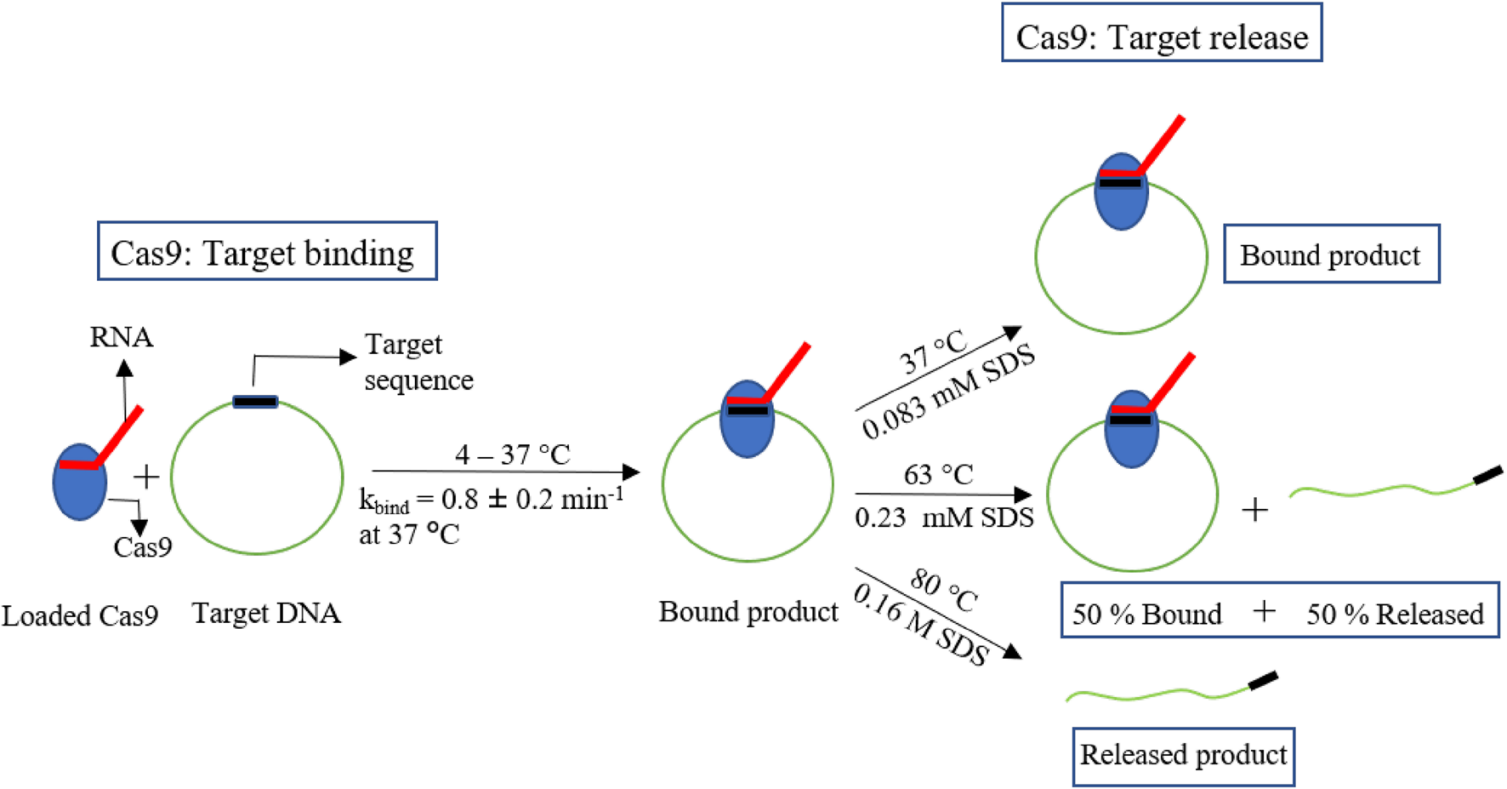
Schematic of Cas9 binding and release. Our data quantifies high temperature and SDS denaturant as two efficient pathways for Cas9 release of cleaved DNA. The green circle represents the target DNA and solid black rectangle on the target DNA denotes the target sequence, red line is the guide RNA and the solid oval blue represents the Cas9. We show that the Cas9.RNA complexes can efficiently bind to target DNA at temperature as low as 4 °C. The enzyme complex binds strongly to the cleaved DNA ends and can be released with high temperature (80 °C) or SDS denaturant (0.16 M).

Next, we studied the temperature dependence of Cas9 binding in 4–25 °C temperature range. Most of the previous studies on Cas9 functionality assays^19,27^ as well as the other gene editing enzymes such as Cas12a (CpfI)^28^ were confined to temperatures of 30 °C and above. From our experiments, we show that Cas9 remains functionally active even at temperatures as low as 4 °C and could very efficiently bind and cleave the target. This demonstrates the possibility of performing temperature-sensitive experiments with Cas9 which is an important aspect in genome editing^19,29^. Next, we showed temperature and SDS-dependent release of the cleaved products and the Cas9 cleaved target DNA remains stably bound to the enzyme and is released only in a temperature or denaturant dependent manner. The schematic in Fig. 5 summarizes our results of time and temperature dependent Cas9–DNA binding and release patterns with both temperature and SDS.

Upon mixing RNA loaded Cas9 and target DNA, the reaction step involving searching and binding to target progressed very fast. We found that at 37 °C or 0.083 mM SDS, the Cas9–DNA complex remained in a bound state and released 50% of cleaved products only when the temperature was increased to 63 °C (336 K) or SDS concentration was increased to 0.23 ± 0.04 mM. The cleaved products were completely (≥ 85%) released when the temperature was further increased to 80 °C or when SDS concentration was increased to 0.16 M. It is known that the Cas9 interaction with DNA involves DNA-RNA hybridization accompanied with structural changes in Cas9. It means that the DNA can be released from Cas9 either by agitating the DNA by increasing thermal energy that lets it escape from the enzyme or by denaturing the Cas9 thereby letting it release the cleaved DNA ends. Our study describes breaking the Cas9–DNA interaction via temperature and SDS denaturant based pathways and quantifies the two product-release mechanisms. We foresee applications of our results to multiple scenarios of temperature dependent control of Cas9 based genome editing, in both in vivo and in vitro conditions. Recently, it was observed that the efficiency of genome editing in *X. laevis* is improved upon incubating the embryos at low-temperature after injecting the CRISPR–Cas9 components^30^. Our observations on high efficiency of Cas9 binding to target at very low temperatures provides opportunities to edit genomes of the less explored cryophiles which have an optimal growth temperature of 15 °C^31^. The temperature dependent studies on binding and release of DNA by Cas9 reveals the energetics of these processes which is important for performing comparative studies that are essential to achieve efficient genome engineering.

## Supplementary Information

Supplementary Information.

## Data Availability

All data generated or analysed during this study are included in this published article and its supplementary information file.
