# Supplementary material for: Temperature dependent in vitro binding and release of target DNA by Cas9 enzyme
Source: Sci Rep. 2022 Sep 9;12:15243. doi: 10.1038/s41598-022-19485-x (PMC9463129; doi:10.1038/s41598-022-19485-x)
Supplement: Supplementary file 1 — Supplementary Information. [file 41598_2022_19485_MOESM1_ESM.docx]

**Temperature dependent *in vitro* binding and release of target DNA by Cas9 enzyme**

Serene Rose David^#^, Sumanth Kumar Maheshwaram^#^, Divya Shet, Mahesh B. Lakshminarayana & Gautam V. Soni^*^

Raman Research Institute, Bangalore, Karnataka – 560080. INDIA

**Supplementary Information:**

This file contains following sections:

(1) FIGURES: S1 to S5
(2) SUPPLEMENTARY METHODS SECTIONS: S1 to S2
(3) RAW FULL-LENGTH GEL IMAGES GENERATED IN THIS STUDY: Rawdata 1 to Rawdata 15

**FIGURES:**

Figure S1: **Cas9 Bound and released products of gel purified conformations of target DNA**. The purified supercoiled & nicked circular DNA was obtained by gel purification using QIAquick gel extraction kit (Qiagen). Lanes 2, 7 and 13 show the gel purified supercoiled, regular amplified supercoiled and 50:50 mix of gel purified supercoiled and gel purified nicked circular DNA respectively. Lanes 3, 8 and 14 show the target DNA when incubated with apo-Cas9. Lanes 4, 9 and 15 show the shift of the target DNA when bound to Cas9.RNA. Lanes 5, 10 and 16 show the release of cleaved products when heated to 90 °C. Lanes 6, 11 and 17 show the release of cleaved products by SDS. Lanes 1 & 12 are 1 kb DNA ladder.

Figure S2: **Time dependence of Cas9 binding to target DNA.** The gels 1, 2 and 3 show the repeated experiments performed to quantify the rate of Cas9 binding to target DNA from the unbound fraction that correspond to Figure 2B. In all gels, lane 1 is the 1kb ladder, lane 2 corresponds to control DNA, lane 3 correspond to DNA+ apo-Cas9 at 37 °C. Lane 4 correspond to the Cas9 bound to target DNA at 37 °C for 60 min and acts as the binding control. The lanes 5 to 16 correspond to the bound fraction at 0 s,4s, 10s, 30s, 40s, 50s, 1 min,2 min, 3 min, 10 min, 30 min and 60 min respectively. Lane 17 corresponds to the SDS release of the product at 0 min. Gels 1 is reproduced from Fig 2A.

Figure S3A: **Temperature dependence of Cas9 binding the target DNA**. The percentage of Cas9 bound DNA at temperatures from 4 °C, 15 °C, 25 °C, 35 °C, 37 °C and 45 °C was quantified from the above two gels that correspond to Fig 3C. In gel 1, lane 1 is control DNA, lane 2 is the 1 kb ladder, lane 3 is DNA+apo-Cas9 at 37 °C and lane 4 is DNA+apo-Cas9 at 90 °C. In gel 2, lane 1 is the 1 kb ladder, lane 2 corresponds to control DNA, lanes 3 and 4 corresponds to DNA+apo-Cas9 at 37 °C and 90 °C respectively. The lanes from 5 to 16 remains the same for both the gels. Gel 2 is reproduced from Fig 3A.

Figure S3B: **Temperature dependence of Cas9 release of cleaved target DNA**: The percentage of released DNA at temperatures from 37 °C, 50 °C, 60 °C, 70 °C, 80 °C, 90 °C and 100 °C was quantified from the above three gels that corresponds to Fig 3D. Lanes 1 and 12 are 1 kb ladder. Lane 2 is control DNA, lanes 3 and 4 correspond to DNA+ apo-Cas9 at 37 °C and 90 °C respectively. Lanes 5 – 11 show the DNA released at temperatures 37 °C, 50 °C, 60 °C, 70 °C, 80 °C, 90 °C and 100 °C respectively. Gel 3 is reproduced from Fig 3B.

**Figure S4: Temperature stability of linear 3 kb DNA.** In Gel 1, we show, in triplicate, DNA stability post Cas9 cleavage and then incubated for 10 minutes at different temperatures. Lanes 1 and 16 are 1 kb ladder. Lane 2 is control supercoiled DNA, Lane 3 has DNA+ apo-Cas9 (without RNA) at 37 °C. Lanes 4 (8 and 12) correspond to sample at 37 °C. Lanes 5 (9 and 13), 6 (10 and 14) and 7 (11 and 15) correspond to the product release at 80 °C, 90 °C and 100 °C, respectively. Gel 2 shows the target scDNA linearized by three different enzymes: Cas9, ScaI and NotI. Lanes 1 and 16 are 1 kb ladder. Lane 2 is control scDNA, Lane 3 corresponds to DNA+ apo-Cas9 (without RNA) at 37 °C. Lane 4 corresponds to sample at 37 °C. Lanes 5, 6 and 7 correspond to the product release by Cas9 at 80 °C, 90 °C and 100 °C respectively. Lanes 8 to 11 and lanes 12 to 15 correspond to the products after restriction digestion by ScaI and NotI and then incubated for 10 mins at 37 °C, 80 °C, 90 °C and 100 °C respectively. Both in Gels 1 and 2, we see degradation of linear 3 kb DNA at 100 °C.

Figure S5: **Quantification of SDS-based release of Cas9 cleaved products.** The SDS based release of target DNA for different concentrations of SDS was quantified from the above 3 gels. Lanes 1 and 17 correspond to 1 kb ladder. Lane 2 is control DNA, lanes 3 is DNA+ apo-Cas9 at 37 °C and lane 4 is DNA+Cas9.RNA at 37 °C. Lanes 5 – 16 show the release of cleaved products at final concentrations of SDS - 0.083 mM, 0.10 mM 0.21 mM, 0.41 mM, 0.83 mM, 8.3 mM, 16 mM, 32 mM, 58 mM, 0.16 M, 0.29 M and 0.50 M. respectively. Gel 2 is reproduced from Fig 4A.

**METHODS**

**S1: Steps for Gel Image Processing**^1^

1. **Download Program:** Download the ImageJ program from http://rsb.info.nih.gov/ij.
2. **Open Image:** Open the raw image from the file > open.
3. **Crop Image:** To crop the image go to Image > crop. Crop the image according to the required size.
4. **Convert to grey scale image:** Go to image > Type > 8 bit.
5. **Level Image:** If the image does not look exactly horizontal, we can level the image using the menu Image > Transform > Rotate.

A dialog box will appear that allows you to rotate the image by an Angle (degrees).

Input positive values for clockwise rotation and negative values for anticlockwise direction. You can tick the Preview option if necessary to see the rotation, set the Angle to zero and increase the grid values. You can then compare your lanes with the horizontal and vertical lines of the grid. Change the angle value until the lanes are horizontally levelled.

6. **Invert Image:** Then go to Edit > Invert
7. **Adjust Brightness/Contrast:** To change the brightness and contrast use the menu Image > Adjust > Brightness.

A pop-up window will appear as shown below where you can make appropriate changes in your image. Once done click the Apply button.

8. **Save Image:** To save image in your specific folder, go to menu File > Save As > Tiff/Jpeg etc.

The above steps were followed to display the gel images in the manuscript and supplementary information file.

**S2: Steps for Gel band quantification**

1. Follow the steps 1 to 5 of the procedure in Section S1.
2. Choose any one of the bands in the gel.
3. Select the rectangular tool from the menu File.

4. Draw a box with a dimension in such a way that it should contain the entire gel band.
5. Use this box for the background as shown below. You can either place the box above or below the band to be quantified.

6. To measure the intensity of the gel region in the box go to menu Analyse > Measure.

A pop-up window will appear showing the results as follows:

7. This ‘Mean’ value showed is regarded as the mean background intensity.
8. Now keep the same box again on the band to be quantified.

9. Go to Analyse > Measure. This gives the mean intensity of the band.
10. Proceed with all the bands to be analysed in the same way.
11. Copy and paste the measured values in Microsoft Excel.
12. For each band, multiply the area with its mean value to get the integral intensity value.
13. Subtract the respective background integral from signal integral to get the actual intensity of the particular band.
14. Add the intensities of all the bands in a particular lane in order to get the total intensity value of all the samples added in a particular well.
15. Estimate the percentage of bound (released) DNA according to the following equation:

$\% Bound \left( released \right) DNA =\frac{Actual intensity of bound \left( released \right) DNA}{Total intensity of all bands}*100$

The above steps were followed to quantify the gel images in the manuscript.

**SUPPLEMENTARY REFERENCES**

1.	Mulcahy, D. G. *et al.* Greater than X kb: A quantitative assessment of preservation conditions on genomic DNA quality, and a proposed standard for genome-quality DNA. *PeerJ* (2016) doi:10.7717/peerj.2528.

**RAW FULL-LENGTH GEL IMAGES GENERATED IN THIS STUDY**

Rawdata 1: Full raw image of Fig 1A.

Rawdata 2: Full raw image of Fig 2A and Gel 1 of Fig S2.

Rawdata 3: Full raw image of Fig 3A and Gel 2 of Fig S3A.

Rawdata 4: Full raw image of Fig 3B and Gel 3 of Fig S3B.

Rawdata 5: Full raw image of Fig 4A and Gel 2 of Fig S5.

Rawdata 6: Full raw image of Fig S1.

Rawdata 7: Full raw image of Gel 2 of Fig S2.

Rawdata 8: Full raw image of Gel 3 of Fig S2.

Rawdata 9: Full raw image of Gel 1 of Fig S3A.

Rawdata 10: Full raw image of Gel 1 of Fig S3B.

Rawdata 11: Full raw image of Gel 2 of Fig S3B.

Rawdata 12: Full raw image of Gel 1 of Fig S4.

Rawdata 13: Full raw image of Gel 2 of Fig S4.

Rawdata 14: Full raw image of Gel 1 of Fig S5.

Rawdata 15: Full raw image of Gel 3 of Fig S5.
